# Adenosine Deaminase Activity Is a Sensitive Marker for the Diagnosis of Tuberculous Pleuritis in Patients with Very Low CD4 Counts

**DOI:** 10.1371/journal.pone.0002788

**Published:** 2008-07-30

**Authors:** Kamaldeen Baba, Anwar A. Hoosen, Nina Langeland, Anne M. Dyrhol-Riise

**Affiliations:** 1 Institute of Medicine, University of Bergen, Bergen, Norway; 2 Center for International Health, University of Bergen, Bergen, Norway; 3 Department of Microbiological Pathology, University of Limpopo, Limpopo, South Africa; 4 Department of Medicine, Haukeland University Hospital, Bergen, Norway; Duke University, United States of America

## Abstract

**Background:**

Adenosine Deaminase Activity (ADA) is a commonly used marker for the diagnosis of tuberculous pleural effusion. There has been concern about its usefulness in immunocompromised patients, especially HIV positive patients with very low CD4 counts. The objective of this study was to evaluate the sensitivity of ADA in pleural fluid in patients with low CD4 counts.

**Materials and Methods:**

This was a retrospective case control study. Medical files of patients with tuberculous pleuritis and non-tuberculous pleuritis were reviewed. Clinical characteristics, CD4 cell counts in blood and biochemical markers in pleural fluid, including ADA were recorded.

**Results:**

One ninety seven tuberculous pleuritis and 40 non- tuberculous pleuritis patients were evaluated. Using the cut-off value of 30 U/L, the overall sensitivity, specificity, positive likelihood ratio, and negative likelihood ratio of ADA was 94%, 95%, 19, and 0.06 respectively. The mean CD4 cell counts among TB pleuritis patients was 29 and 153 cells/microL in patients with CD4 <50 cells/microL and >50 cells/microL, (p<0.05) respectively. The corresponding mean ADA values for these patients were 76 U/L and 72 U/L respectively (p>0.5). There was no correlation between ADA values and CD4 cell counts (r = −0.120, *p* = 0.369).

**Conclusion:**

ADA analysis is a sensitive marker of tuberculous pleuritis even in HIV patients with very low CD4 counts in a high TB endemic region. The ADA assay is inexpensive, rapid, and simple to perform and is of great value for the immediate diagnosis of tuberculous pleuritis while waiting for culture result and this has a positive impact on patient outcome.

## Introduction

Tuberculous pleuritis occurs in about 25% of total tuberculosis (TB) cases in South Africa [1], [2]. Rapid diagnosis and prompt treatment is needed to reverse the morbidity and mortality due to TB. The diagnosis of tuberculous pleuritis remains a challenge because of the small numbers of bacilli in the pleural fluid [3], [4], [5]. Pleural fluid staining for acid fast bacilli (AFB) and culture has low sensitivity [4] and culture takes more than a week even when using automated systems. Pleural biopsy culture and typical histology of necrotizing granuloma is more sensitive than pleural fluid culture [4]. However, the invasiveness of the procedure, inability to get representative pleural tissue and the risk of complications are limitations in obtaining pleural biopsies. We have recently demonstrated that immunohistochemistry with the anti- MPT64 antibody as diagnostic tool offers a reasonable high sensitivity for the diagnosis of TB pleuritis [6].

A well established biological marker for diagnosis of tuberculous pleuritis is Adenosine Deaminase Activity (ADA) [4], [7], [8], [9], [10]. The ADA assay principle is based on the detection of either hydrogen peroxide or ammonia after enzymatic deamination of adenosine to inosine by ADA. The unit cost of different commercial assays in South Africa is about South African rands nine (equivalent to U.S dollars 1.2). Production of the enzyme Adenosine Deaminase in pleural fluid reflects the presence of activated T lymphocytes and monocytes [11]. Therefore, it is expected that ADA will be lower in HIV co-infected patients and/or other immunocompromised patients with low blood CD4 counts. It has been shown that ADA values correlate with CD4 counts in the pleural fluid of TB patients [12]. Hsu *et al* [*13*] has shown lower sensitivity of ADA for the diagnosis of TB pleural effusion in 10 immunocompromised patients with chronic illness such as diabetes, cirrhosis, renal failure and leukaemia. In contrast, Riantawan *et al* [*14*] did not find any difference in the performance of ADA in 37 HIV positive patients with mean CD4 counts above 100 cells/microL. A recent study by Reuter *et al* [*15*] reported low sensitivity of ADA in tuberculous pericarditis patients with advanced HIV disease and median CD4 count of 59 (6–115) cells/microL. CD4 counts below 50 are regarded as very low and are an indication for disease progression [16]. However, it is not known how the ADA will perform for the diagnosis of tuberculous pleuritis, a common extra-pulmonary location in severely immunocompromised HIV patients. The aim of this study was to evaluate the sensitivity of ADA in tuberculous pleuritis patients with low CD4 counts and to determine whether there was any correlation between CD4 cell counts and ADA values.

## Materials and Methods

Patients were identified through tracing of pleural fluid specimens sent for culture to the microbiology laboratory at the Dr George Mukhari (DGM) Hospital Ga-Rankuwa, Pretoria, South Africa. All patients presenting with pleural effusion had pleural tap and subsequently pleural fluid examination. Patients were put on TB treatment after pleural tap if there was strong suspicion of TB based on clinical features such as, history of contact with TB patients, TB history, chronic cough, night sweats, fever and loss of weight.

The diagnosis of TB pleuritis was confirmed if culture of *Mycobacterium tuberculosis* in pleural fluid or tissue was positive or there was caseous granuloma in the pleural biopsy and was probable if; (1) there were clinical features such as history of TB contact, past history of TB, chronic cough, night sweats, fever and loss of weight; and (2) had clinical diagnosis of TB and started on TB treatment in the absence of any other cause of effusion. The control non- TB pleuritis group include; (1) malignant pleural effusion confirmed by cytological examination of pleural fluid/histological examination of pleural biopsy; (2) Pleural effusion of other known etiology such as congestive cardiac failure or para-pneumonic effusion. Cases without ADA results were excluded from the analysis.

The clinical records of patients as described above for the period January 2004 to January 2007 were reviewed. Data regarding clinical presentation (cough, fever, chest pain, night sweats, loss of weight, diarrhoea, oral thrush), acid fast staining (AFB) and culture results of pleural fluid, HIV status, blood CD4 cell counts, and biochemical parameters in pleural fluid (ADA, lactate dehydrogenase , total protein) were collected. The ADA was analysed using a commercial colorimetric assay kit (Diazyme General Atomics, CA). The cut-off value for positive ADA result was 30 U/L according to clinical practice at this hospital [17]. The patients were stratified according to the CD4 count as follows, <50 and >50, cells/microL as referred above [16].

The study was evaluated and approved by the Research, Ethics and Publications Committee at the DGM Hospital, University of Limpopo, South Africa and the Regional Committee for Ethics in Medical Research in Bergen, Norway.

### Statistical analysis

Data analysis was made by SPSS (SigmaStat; SPSS Inc; Chicago, IL). Mean, correlation coefficient, and group comparison was made with non parametric Mann-Whitney U test.

## Results

A total of 300 pleural fluid samples were submitted to the laboratory for the diagnosis of TB over the 3 year study period of which 237 with available ADA results were included in the study. There were 197 TB pleuritis patients: mean age 35 years with 107 females and 40 non-TB pleuritis patients: mean age 50 years with 19 females. Among the TB pleuritis patients, 72 were confirmed TB pleuritis while the non-TB pleuritis included 26 malignancies (adenocarcinoma, mesothelioma, squamous cell carcinoma, metastatic liver carcinoma, bronchial carcinoma and Kaposi sarcoma), 10 congestive cardiac failures, 2 hydrothorax, 1 *Streptococcus pneumoniae* effusion and 1 *Klebsiella pneumoniae* empyema. The mean ADA for each category is shown in Table 1. The ADA value was positive, above the cut-off of 30 U/L in 186 of the 197 TB patients giving a sensitivity of 94% (Table 2). The mean ADA value was 71.2U/L whilst the mean total protein value was 51 g/dl.

**Table 1:** The mean ADA values in the different diagnostic category.

Cases	Mean ADA (U/L) ±SD
Malignancy* (N = 26)	12.9±13.1
Congestive heart failure (N = 12)	7.16±6.2
Streptococcus pneumoniae effusion (N = 1)	11
Klebsiella pneumoniae empyema (N = 1)	200
Total TB pleuritis (N = 197)	71.2±39.6

SD = standard deviation;

* Kaposi sarcoma is the only malignancy with ADA of 73 above the cut-off.

**Table 2:** Validity of ADA as a diagnostic test.

Cases	Sensitivity % (CI)	Specificity % (CI)	PPV % (CI)	NPV (%) (CI)	LR+ (CI)	LR− (CI)
Confirmed TB pleuritis (n = 72)	94 (86–98)	95 (82–99)	97 (89–99)	90 (76–97)	18.9 (4.9–73	0.06 (0.02–0.15)
Clinical TB (n = 125)	94 (88–97)	95 (82–99)	98 (93–100)	84 (70–93)	18.9 (4.9–73.0)	0.06 (0.03–0.12)
Total TB Pleuritis (n = 197)	94 (90–97)	95 (82–99)	99 (96–100	77.5 (63–88)	18.9 (4.9–72.9)	0.06 (0.03–0.10)

PPV = positive predictive value; NPV = negative predictive value; CI = 95% confidence interval; LR+ = positive likelihood ratio; LR− = negative likelihood ratio.

Among the 125 probable TB pleuritis patients; 89 were HIV positive, 18 were HIV negative and HIV was not available for 18 patients. The median ADA values were 64 U/L and 58 U/L for HIV positive and HIV negative respectively.

CD4 cell counts were available for 58 of the 72 patients among the confirmed TB pleuritis patients. Fifty-one (88%) of the patients with known CD4 cell counts were confirmed HIV positive, whereas two were HIV negative with CD4 cell counts of 482 and 512 cells/microL. For five of the TB pleuritis patients the HIV was not documented, but as the patients had CD4 cell counts in the range of 19–208 cells/microL they were most likely HIV positive.

The ADA values with corresponding CD4 cell counts <50 and >50 cells/microL among confirmed TB pleuritis patients are presented in table 3. The mean CD4 cell counts in the confirmed TB pleuritis cases was 26 cells/microL in patients with CD4<50 cells/microL and 158 cells/microl in patients with CD4 count >50 cells/microL, (p<0.05) and the corresponding values among total TB cases was 29 and153 (p<0.05) respectively.

**Table 3:** HIV status, CD4 counts and ADA values for the 2 groups with <50 (N = 25) and >50 (N = 33) CD4 counts among confirmed TB pleuritis patients.

	CD4<50 cells/microL				CD4 >50 cells/microL		
Case	HIV	CD4	ADA		HIV	CD4	ADA
1	pos	1	46	1	pos	55	38
2	pos	2	55	2	pos	55	60
3	pos	4	41	3	pos	57	60
4	pos	7	86	4	pos	60	112
5	pos	13	4	5	pos	64	112
6	pos	12	98	6	NA	65	61
7	pos	15	200	7	pos	66	133
8	pos	18	21	8	pos	66	51
9	pos	19	42	9	pos	70	36
10	pos	19	210	10	pos	74	112
11	NA	19	55	11	pos	75	73
12	pos	20	71	12	pos	77	43
13	pos	30	52	13	pos	79	53
14	pos	32	56	14	pos	80	186
15	pos	33	51	15	pos	80	38
16	NA	33	55	16	pos	105	1
17	pos	35	132	17	pos	115	88
18	pos	38	124	18	pos	121	44
19	pos	40	48	19	pos	132	53
20	pos	40	112	20	pos	163	64
21	pos	40	93	21	pos	168	35
22	pos	41	70	22	pos	179	44
23	pos	43	200	23	NA	180	38
24	pos	45	71	24	pos	200	107
25	pos	46	32	25	pos	200	49
				26	NA	208	67
				27	pos	229	96
				28	pos	241	113
				29	pos	251	102
				30	pos	302	100
				31	pos	401	35
				32	neg	482	70
				33	neg	512	28

pos = positive, neg = negative, NA = not available, ADA = adenosine deaminase.

**Figure 1:**
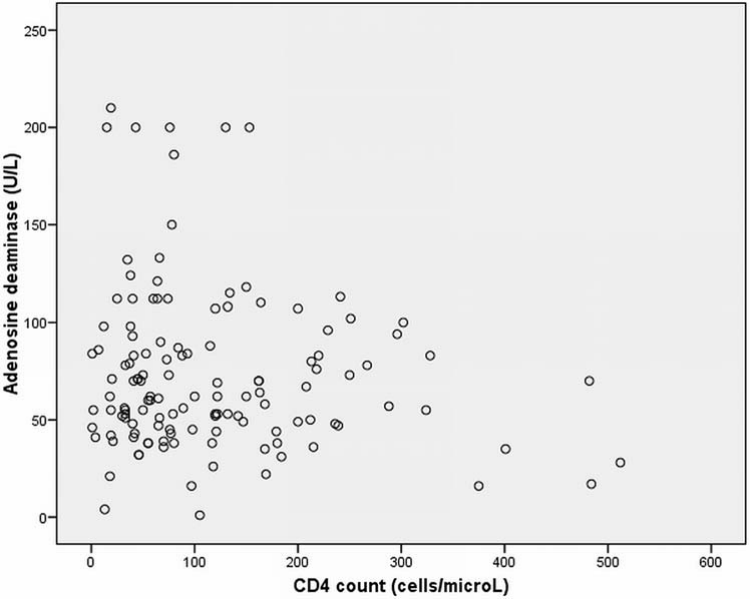
Correlation plot of ADA in pleural fluid and blood CD4 counts in TB pleuritis patients (N = 129).

The sensitivity and specificity of ADA was 94% and 95% respectively as shown in table 2. The positive and negative likelihood ratio was 19 and 0.06 respectively. The mean ADA values for the different categories is shown in table 3. The mean ADA value was highest in tuberculous pleuritis except for Kaposi sarcoma, and empyema in which the ADA gave false positive result. The sensitivity of ADA was not affected by low CD4 cell count. In addition, there was no significant difference in the mean ADA value in patients with lower CD4 counts (81 U/L±55.1); (76 U/L±45.3) compared to those with higher CD4 cell counts (70 U/L±37.8); (72 U/L±40.6), (p>0.5) among confirmed and total TB pleuritis respectively. The four patients with CD4 cell counts of less than 10 cells/microL (1, 2, 4, and 7 cells/microL) among confirmed TB pleuritis patients had ADA values above the cut-off with values of 46, 55, 41, and 86 U/L, respectively (Table 3). There was no correlation between ADA values and blood CD4 cell counts (Figure 1).

There was no statistical difference in the absolute blood lymphocyte count; pleural fluid lymphocytes number or percentage, lactate dehydrogenase and total protein between patients with CD4 cell counts <50 and >50 cells/microL when group comparison was made with non parametric Mann-Whitney U test (Table 4).

**Table 4:** Group comparison of laboratory markers among total TB pleuritis patients with <50 (N = 41) and >50 (N = 88) CD4 counts.

Laboratory test (N)	Mann-Whitney U values	p- values
**In pleural fluid**		
Adenosine deaminase U/L (129)	1725.5	0.691
Lymphocyte cells/microL (86)	586.0	0.060
Lymphocyte % (68)	401.5	0.216
Lactate dehydro-genase U/L (115)	1315.0	0.325
Total protein g/L (105)	1211	0.925
**In blood**		
CD4 count cells/microL (129)	<0.05*	<0.05*
Absolute Lymphocyte count X10^9^/L (75)	552.0	0.181
Red blood cells X 10^12^/L (111)	1044.5	0.054

* = significant difference between the 2 groups.

Furthermore there was no correlation between ADA values and blood CD4 counts, absolute lymphocyte count and red cell counts in blood or lymphocyte counts and percentage, lactate dehydrogenase and total protein in pleural fluid (Table 5).

**Table 5:** Correlation (r) values for ADA and other blood and pleural fluid markers in tuberculous pleuritis patients (N = 197).

Test	r	p-values
**In blood**		
CD4 counts cells/microL	−0.14	0.114
Absolute blood lymphocytes x 10 ^9^/L	−0.049	0.667
Red blood cells x 10^12^/L	−0.044	0.644
**In pleural fluid**		
Leucocytes	0.028	0.798
LYM%	0.110	0.373
Lactate dehydro-genase U/L	0.152	0.104
Total protein g/L	0.045	0.649

LYM% = pleural fluid lymphocyte percentage of total leucocytes.

## Discussion

We have evaluated the usefulness of the ADA assay in predominantly HIV positive patients with very low CD4 counts. The overall sensitivity of the ADA assay in diagnosing tuberculous pleuritis was 94%. This finding is similar to that reported in a number of studies [12], [14], [18], [19], [20]. However this is the first study demonstrating high sensitivity of the ADA assay in HIV patients with very low CD4 cell counts. As shown in our study there was no significant difference in sensitivity when comparing cases with CD4 cell counts of less than 50 cells/microL to those with CD4 counts greater than 50 cells/microL. In fact, the mean ADA value was higher in the group with the lower CD4 cell count.

The ADA is useful for the diagnosis of TB pleuritis since the result is available on the same day compared to culture which takes about 2 weeks. This allows for rapid institution of TB treatment leading to improvement in patient outcome[21]. Furthermore the positive likelihood ratio (LR) was >10 and the negative likelihood ratio was <0.1, this implies that the ADA is useful in clinical practice to rule in and rule out TB pleuritis. The advantage of LR is that it is not affected by prevalence of disease as opposed to positive predictive value.

Previous studies evaluating the usefulness of ADA for the diagnosis of tuberculous pleuritis have either a very small number of HIV positive cases [22] or patients with higher mean CD4 counts [14]. Hsu *et al* [13] have shown a low sensitivity for ADA in tuberculous pleural effusion in immunocompromised chronic diseases patients, however only 10 patients were evaluated and the HIV status was not determined. Corral *et al* [*23*] reported a similar low sensitivity in HIV positive tuberculous meningitis cases whilst Reuter *et al* [*15*] reported low sensitivity of ADA in tuberculous pericarditis patients with advanced HIV disease compared to cases with higher CD4 cell count. The difference in the outcome of our retrospective study compared with that of Reuter *et al* may be due to the fact that majority of the patients in this study had lower CD4 cell counts.

Different cut-off values ranging from10 to 71 U/L have been used for positive ADA test, but the type of ADA assays has not shown any variations in sensitivity [24]. The cut-off value of 30 U/L for tuberculous pleuritis as used in this study is expected to offer a specificity of 98% [17] and is used in the clinical setting at our institution and by most laboratories in South Africa.

We did not find any correlation between the ADA values and CD4 cell counts. Even at CD4 counts of less than 10cells/microL, the ADA values were still above the cut-off. We also did not find any correlation between ADA values and CD4 cell counts in another cohort of South African HIV infected TB pleuritis patients [25]. This may be due to higher immune activation in HIV positive patients with very low CD4 counts [26]. Furthermore, we did not find any correlation between ADA values and pleural fluid lymphocyte numbers or percentage in agreement with the study by Lee *et al* [*27*] *,* but in contrast to other studies [12], [28]. One explanation could be that the isoenzyme ADA-2 which contributes significantly to total ADA in diagnosing TPE is found mainly in the monocytes [29] which are not significantly affected in HIV patients compared to CD4 T-lymphocytes.

Interferon gamma is considered to be a better marker than ADA for the diagnosis of tuberculous pleuritis, since its estimation is not affected by immununosuppression [30], [31]. However, in our study the ADA assay performed well even at very low CD4 count below 10 cells/microL and the cost of estimating ADA is 20 times less than that of interferon gamma estimation. Therefore ADA analysis is more cost effective than the estimation of interferon gamma for the diagnosis of tuberculous pleuritis in developing countries [18].

We have shown that ADA offers a high sensitivity and is rapid for the diagnosis of tuberculous pleuritis in HIV patients with low CD4 counts in a high TB endemic region. This is an important finding for countries with high burden of TB and HIV co-infection. Furthermore ADA is inexpensive, rapid, and simple to perform and is of great value in the diagnosis of tuberculous pleuritis for early institution of TB treatment.
